# The Plasticity of Extinction: Contribution of the Prefrontal Cortex in Treating Addiction through Inhibitory Learning

**DOI:** 10.3389/fpsyt.2013.00046

**Published:** 2013-05-30

**Authors:** J. T. Gass, L. J. Chandler

**Affiliations:** Department of Neurosciences, Medical University of South Carolina, Charleston, SC, USA

**Keywords:** extinction learning, prelimbic, infralimbic, prefrontal cortex, addiction

## Abstract

Theories of drug addiction that incorporate various concepts from the fields of learning and memory have led to the idea that classical and operant conditioning principles underlie the compulsiveness of addictive behaviors. Relapse often results from exposure to drug-associated cues, and the ability to extinguish these conditioned behaviors through inhibitory learning could serve as a potential therapeutic approach for those who suffer from addiction. This review will examine the evidence that extinction learning alters neuronal plasticity in specific brain regions and pathways. In particular, subregions of the prefrontal cortex (PFC) and their projections to other brain regions have been shown to differentially modulate drug-seeking and extinction behavior. Additionally, there is a growing body of research demonstrating that manipulation of neuronal plasticity can alter extinction learning. Therefore, the ability to alter plasticity within areas of the PFC through pharmacological manipulation could facilitate the acquisition of extinction and provide a novel intervention to aid in the extinction of drug-related memories.

Once believed to result from an immoral personality or lack of will power, it is now clear that drug addiction is a disease of the nervous system that involves uncontrollable drug intake and compulsive drug-seeking behavior. As such, addiction is characterized by periods of repeated drug use followed by unsuccessful attempts to maintain abstinence. As a chronic relapsing disorder, addiction is associated with numerous brain changes that include signaling pathways, neurotransmitters, and cell mechanisms that overlap with those that mediate normal learning and memory processes. Thus, there have been numerous theories that incorporate mechanisms of learning and memory as a basis for drug addiction (O’Brien et al., 1992; Di Chiara, 1999; Volkow et al., 2002; Kelley, 2004; Wise, 2004; Hyman, 2005; Weiss, 2005). These theories suggest that through basic conditioning principles, certain behaviors and drug-environment associations become “overlearned” and thus contribute to the compulsive behavior of addicts.

In classical Pavlovian conditioning, also referred to as stimulus-outcome conditioning, the presentation of a conditioned stimulus (CS) paired with presentation of an unconditioned stimulus (US) after repeated pairings comes to elicit a conditional response (CR). In a drug context, the repeated pairing of the CS (e.g., environmental cues) with the reinforcing properties of a drug (US) results in the ability of the CS alone to elicit drug-seeking behaviors. Conversely, instrumental conditioning, also referred to as response-outcome conditioning, involves learning through consequences (either positive or negative) that are contingent upon a particular behavior. In a drug context, behaviors that lead to the reinforcing effects of a drug are more likely to be repeated in the future. It is believed that drug-taking behaviors become compulsive and automatic (instrumental conditioning) with repeated drug exposure, and the associations between drugs and specific environmental cues and context become overly salient (classical conditioning). Conditioning processes also play a role in the influence of environments that predict drug availability to induce craving and promote relapse (Childress et al., 1988, 1999; Kalivas and Volkow, 2005).

The ability to suppress drug-seeking behaviors that are heavily influenced by drug memories is a logical therapeutic approach in the prevention of relapse. Extinction is the gradual reduction of a CR when the CS is no longer paired with the US. Functionally, it is observed as a decrease in responding from higher levels observed prior to extinction to lower levels following extinction training. Theoretically, this type of inhibitory training could reduce the occurrence of behaviors that are trademarks of addiction including drug-seeking and relapse. However, current implementations of extinction-based techniques, such as exposure therapy, have a poor record of efficacy (Childress et al., 1993; Conklin and Tiffany, 2002a,b). Therefore, there is a need to better understand the neural mechanisms that underlie extinction learning and develop therapeutic interventions that increase the success rates of cue exposure therapies. This could lead to treatments involving a combination of behavioral training and pharmacological interventions that create a more robust and persistent decrease in cue-induced affective responses to drug memories (Davis et al., 2006). A substantial amount of research has focused on the neurobiological processes that underlie the extinction of conditioned fear and non-drug reinforcers (e.g., food). While the majority of previous work has focused on understanding the mechanisms involved in fear/non-drug extinction, there is an increasing interest in understanding how these principles apply to addiction related behaviors. Results from the fear and non-drug extinction field have greatly informed and helped guide studies in addiction. Therefore, while the focus of this review is on the extinction of drug-seeking behavior, observations from the fear/non-drug extinction field will be incorporated where appropriate.

## What is Extinction Learning?

At first glance, the phenomenon of extinction may appear to simply represent a process that involves the unlearning, forgetting, and/or erasure of a previously formed memory (Rescorla and Wagner, 1972). However, a large body of evidence gained over the past several decades provides strong support for the idea originally suggested by Pavlov (1927) that extinction is “new” and “active” learning and is not simply the “unlearning” or erasure of previously formed associations. Many of these studies have been carried out in rodents and involve the extinction of responding for a natural reinforcer such as food. In contrast, studies of extinction learning in addiction typically involve extinction of self-administration of a drug of abuse such as cocaine. These experimental procedures incorporate aspects of both instrumental and classical conditioning to train animals to perform a behavior (e.g., lever pressing) to receive access to a drug and associate discrete cues (e.g., auditory and/or visual) with the drug’s reinforcing effects. Regardless of the type of reinforcer used (e.g., food or drug), extinction is defined in this review as the omission of a previously delivered unconditioned stimuli/reinforcers or the absence of a contingency between a response and reinforcer (Lattal and Lattal, 2012). In addition, while extinction behavior can be observed in both classical and instrumental conditioning paradigms, this review will not attempt to define the neural mechanisms associated with each form of learning.

The idea that extinction involves new learning has great implications for not only understanding how drug memories can have a lasting influence on relapse but also for the development of pharmacological treatments for addiction. The following lines of evidence from studies examining the extinction of drug-related behaviors support the idea that extinction is indeed new learning:

(1) After extinction training, drug-seeking behavior can be reactivated with a single stimulus without the need for additional behavioral training (Sinha et al., 2000; Stewart, 2000, 2003; Sinha, 2001; Shalev et al., 2002; See, 2005; Epstein et al., 2006; Kalivas et al., 2006; Olmstead, 2006).
(2) Drug-seeking can resume after lengthy periods of abstinence or extinction training indicating that the original drug-memory remains and has not simply been deleted (Hammersley, 1992; Tobena et al., 1993; Corty and Coon, 1995; Di Ciano and Everitt, 2004).
(3) Extinction is context-specific (Bouton, 2000, 2002, 2004; Chaudhri et al., 2008; Wells et al., 2011), which suggests that original memory of drug reinforcement is still present even after extinction training.
(4) The retraining of self-administration after extinction is considerably less compared to original training (Carroll, 1998; Grasing et al., 2005).
(5) Extinction learning has been shown to involve classic cellular hallmarks of learning and memory (Crombag and Shaham, 2002; Sutton et al., 2003; Self and Choi, 2004; Self et al., 2004; Knackstedt et al., 2010).

**Figure 1:**
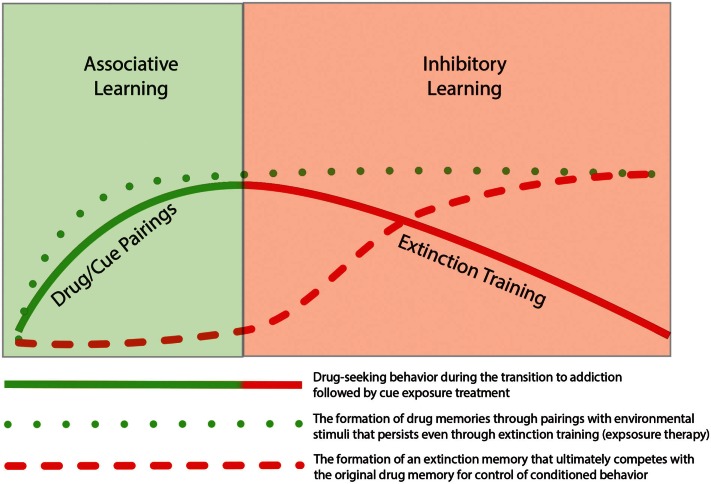
**Depiction of the temporal relationship of associative learning of drug-seeking behavior with inhibitory learning during subsequent extinction of the drug-seeking behavior**. The initial phase of addiction involves associative learning processes in which drug-taking becomes linked through classic Pavlovian conditioning with drug-related cues (e.g., drug paraphernalia or drug-taking environment). With repeated pairing, this association results in formation of a persistent “drug memory.” This memory trace remains long after discontinuation of drug-taking. The extinction of drug-seeking by pairing unreinforced exposure of drug-related cues, does not result in the deletion of the original drug memory, but instead involves the formation of a new inhibitory “extinction memory.” While this new memory provides inhibitory drive over drug-seeking behavior in the short term, the original drug-memory remains, which may explain the high rate of relapse following behavioral extinction therapies.

Thus, findings from the literature on addiction support the idea that extinction training is not the removal of a previously formed association but instead involves the generation of a new memory that competes with the initial memory for control of behavior. As such, the original associative and instrumental conditioning that occurs during the early stages of addiction remains intact. Based on similar findings from the fear extinction literature, Quirk et al. (2006) presented a schematic model to illustrate the idea that even though fear behavior decreases, the original fear memory remains. As depicted in Figure 1 the same concept can be mapped onto the processes of addiction such that drug-seeking behavior declines during extinction training, but the drug-memory remains and competes with the newly formed extinction memory for the control of behavior. The formation of new memories during extinction training likely utilizes neural circuitry involved in basic learning and memory process. In the following sections we review studies that have highlighted specific brain regions and mechanisms involved in extinction learning.

## Neurocircuitry of the Extinction Learning

While the neurocircuitry of extinction is likely diffuse and involves a distributed network, there is evidence for the involvement of several key brain regions in drug-seeking, fear expression, and extinction behavior that could constitute differential circuits associated with each of these behaviors.

### The prefrontal cortex

**Figure 2:**
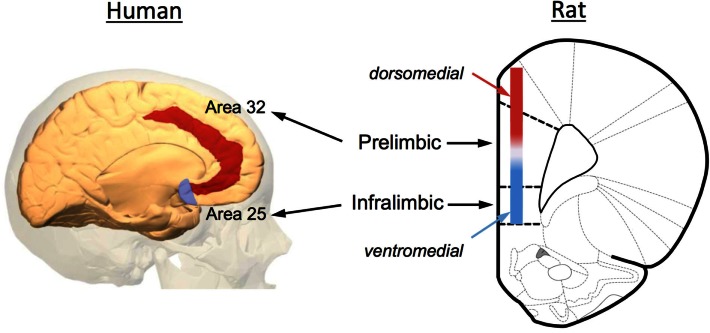
**Anatomical depiction showing the location of the prelimbic (PrL) and infralimbic (IfL) subregions of the medial PFC of the rat and their equivalent regions of the human brain**. Based upon commonality of thalamic inputs, the rodent PrL region is roughly analogous to Brodmann area 32 while the IfL is roughly analogous to Brodmann area 25. Because of the small size of the rodent brain and the lack of defined borders for the PrL and IfL regions, some investigators simply divide the rodent medial PFC into a dorsomedial and ventromedial region as illustrated in the diagram. The original image of the human brain shown on the left was modified from an image downloaded from Wikipedia (http://en.wikipedia.org/wiki/File:Brodmann_area_32_medial.jpg). The original rat brain image shown on the right was modified from Paxinos and Watson (6th Edition).

Increasing evidence has implicated the prefrontal cortex (PFC) in the extinction of both fear and drug-seeking behaviors. Anatomically, the rodent PFC is located in the anterior pole of the frontal cortex and is loosely defined as the anterior cingulate (ACC), medial PFC (mPFC), and orbital frontal cortex (OFC). As illustrated in Figure 2, the rodent mPFC can be further subdivided into a dorsal region called the prelimbic (PrL) cortex and a ventral region called the infralimbic (IfL) cortex. These subregions do not have well demarcated structural boundaries that can often make it difficult to clearly delineate these subregions, especially given the small size of the rodent brain. For this reason, investigators often simply divide this area into a dorsomedial PFC that includes the dorsal region of the PrL cortex and much of the overlying ACC, and a ventromedial PFC that includes the IfL cortex and the ventral portions of the PrL cortex (Figure 2). Defining analogous subregions of the PFC of rodents and human brain is also difficult due to the evolutionary expansion of the PFC. Therefore definitions are based not only upon common anatomical circuitry but also upon function. Based upon similarities in thalamic inputs, the rodent PrL region is considered to be equivalent to Brodmann area 32 (pregenual anterior cortex) and the IfL cortex is equivalent to Brodmann area 25 (subgenual anterior cortex) in the human (Figure 2). It should also be noted that the dorsolateral PFC of humans (conservatively defined as areas 9 and 46) is also considered to be equivalent to the rodent mPFC using a functional definition as both regions are involved in working-memory processes.

**Figure 3:**
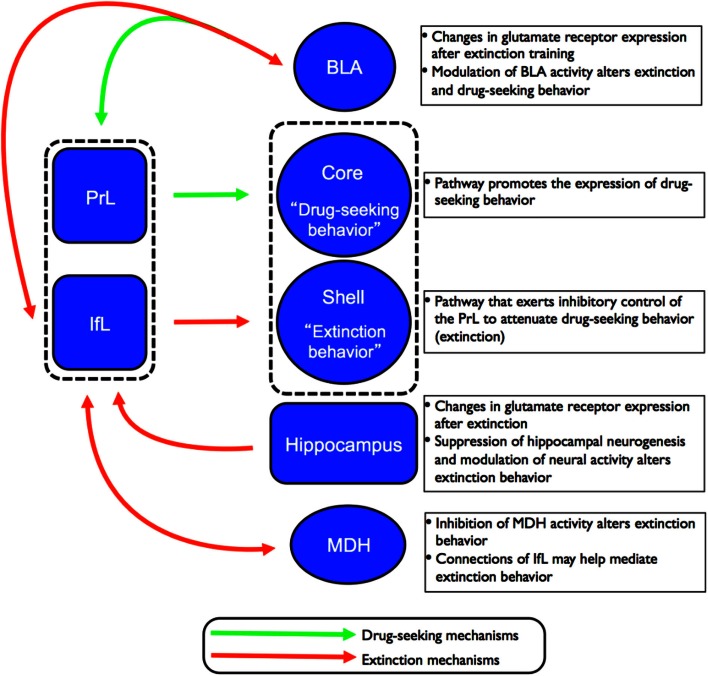
**Schematic of the proposed circuitry involved in the drug-seeking and extinction behavior**. Projections from the PrL cortex to the NAc core regulates the expression of cocaine-seeking behavior (indicated by green arrows) while projections from the IfL cortex to the NAc shell regulates the expression of extinction behavior (indicated by red arrows). Recent studies have also implicated the involvement of other brain regions such as the hippocampus, MDH, and BLA in the neurocircuitry of extinction of drug-seeking behavior.

While complex behaviors such as working memory, impulsivity, motivation, and decision-making have often been linked to the cognitive function of the PFC, a number of recent studies have implicated PFC subregions in extinction behavior. In particular, lesion studies have shown that the PrL cortex is necessary for the expression of conditioned fear while the IfL cortex is critical for the expression of extinction behavior (for reviews, see Quirk et al., 2010; Sierra-Mercado et al., 2011; Milad and Quirk, 2012). Drug-seeking behavior has been studied extensively in humans where it has been shown that presentation of drug stimuli significantly increase activation in specific regions of the PFC (for a review, see Goldstein and Volkow, 2011). Several inactivation studies have also implicated the PrL cortex of the rat as a critical component in the circuitry for drug-seeking behavior including cocaine (McFarland and Kalivas, 2001; Capriles et al., 2003; McLaughlin and See, 2003; McFarland et al., 2004; See, 2005; Di Pietro et al., 2006) and heroin (LaLumiere and Kalivas, 2008; Rogers et al., 2008). Additionally, the IfL cortex, which has been studied extensively in fear extinction, has also been implicated in the extinction of drug-seeking behavior (Ovari and Leri, 2008; Peters et al., 2008a,b). As depicted in Figure 3, converging lines of evidence from both the fear- and drug-conditioning fields suggest that the PrL cortex serves as an “on-switch” for conditioned fear expression and drug-seeking, while the IfL cortex functions as an “off-switch” for the expression of extinction behavior (LaLumiere and Kalivas, 2008; Peters et al., 2008a; Quirk and Mueller, 2008; LaLumiere et al., 2010). These subregions of the PFC could thus serve as candidate regions for plasticity-related changes associated with extinction behavior.

### Dorsal and ventral striatum

Different subregions of the striatum are important for mediating components of reward. The rodent striatum is divided into the dorsal and ventral striatum, and each of these regions can be further subdivided. Due to its involvement in habit learning, the dorsal striatum has been implicated in various aspects of the transition from voluntary behavior to uncontrolled habitual behavior that characterizes drug abuse (Robbins and Everitt, 2002; Weiss, 2005; Izquierdo et al., 2006). In particular, the dorsomedial subregion has been shown to modulate goal-direction actions that transitions to the dorsolateral striatum as these actions become habitual. The ventral striatum or nucleus accumbens (NAc) can be further divided into a lateral “core” and medial “shell” subregion. Through its connections with the PFC, amygdala, hippocampus, and motor regions, the NAc plays a role in guiding emotionally relevant behavioral responses related to the reinforcing properties of drugs and drug-related stimuli (Bonci et al., 2003; Di Chiara and Bassareo, 2007).

Recent studies have also implicated the NAc in extinction of drug-seeking behavior. Cocaine self-administration causes a decrease in tyrosine hydroxylase in the NAc shell which is reversed with extinction training during withdrawal from cocaine (Schmidt et al., 2001). Extinction training also induces an upregulation in the expression of AMPA receptor subunits within the NAc shell (Sutton et al., 2003; Self et al., 2004). More recently, it was shown that inactivation for the NAc shell resulted in the expression of cocaine-seeking behavior, possibly through an interaction with the IfL cortex (Peters et al., 2008a). Similarly, activation of IfL glutamatergic projections with an AMPA receptor positive allosteric modulator reduced cocaine-seeking behavior, and blockade of AMPA activity in the NAc shell attenuated this effect (LaLumiere et al., 2012). Similar findings have been observed with the extinction of ethanol-seeking behavior. For instance, the NAc shell, possibly through interactions with the hypothalamus or the amygdala, helps mediate the expression of extinction behavior (Millan et al., 2010; Millan and McNally, 2011). With regards to the NAc core, extinction training also normalizes cocaine-induced deficits in levels of the GluN1 subunit of the NMDA receptor (Self et al., 2004). Consistent with its role in goal-directed and habitual actions, the dorsal striatum has also been implicated in the extinction of habitual cocaine-seeking behavior (Fuchs et al., 2006). These lines of evidence suggest that there is a significant amount of plasticity that occurs within the dorsal striatum and NAc during extinction learning, and that these regions are central in the neurocircuitry of extinction of drug-seeking behavior.

### Amygdala

As is the case with the PFC and the striatum, the amygdala is made up of a complex of different substructures that differentially contribute to extinction of fear- and drug-seeking behavior. The amydaloid complex includes the basal and lateral subregions (collectively known as the basolateral amygdala, BLA), medial amygdala (MeA), central amygdala (CeA), and cortical amygdala (CoA). The amygdala is involved with various learning and memory processes including formation and consolidation of emotional memories (Cahill et al., 2001; LaBar, 2003). The BLA also has an established role in synaptic plasticity associated with emotion-related behaviors, the processing of emotionally relevant stimuli (Cahill et al., 1995; McGaugh, 2004; Phelps et al., 2004; Maren, 2005; LaBar and Cabeza, 2006), and in stimulus-reward associations (Hatfield et al., 1996; Blundell et al., 2001; Baxter and Murray, 2002; Everitt et al., 2003; See, 2005; Balleine and Killcross, 2006). The BLA also plays an integral role in the formation of associations between drugs and environmental cues (Hiroi and White, 1991; Brown and Fibiger, 1993; Whitelaw et al., 1996; Rizos et al., 2005). While there has been a substantial amount of research implicating the BLA in the extinction of fear conditioning (Myers and Davis, 2002, 2007; Quirk et al., 2010; Sierra-Mercado et al., 2011), studies have also implicated this region in the extinction of drug-seeking behavior. For example, enhancement of glutamatergic transmission within the BLA facilities the extinction of a drug-paired conditioned place preference (CPP) (Shidara and Richmond, 2002; Schroeder and Packard, 2004), and given the essential role of the BLA in drug-seeking (See et al., 2003), it is logical to assume that plasticity within this structure may also influence extinction learning.

### Hippocampus

The hippocampus is known to play an important role in various forms of learning and spatial/contextual memory and in memory consolidation/retrieval (Neves et al., 2008). The hippocampus is also involved in extinction behavior as evidenced by impairments in context-dependent extinction of fear conditioning that results from inactivation of this brain region (Corcoran and Maren, 2001; Corcoran et al., 2005; Ji and Maren, 2005) and cellular substrate inhibition (Szapiro et al., 2003; Vianna et al., 2003; Power et al., 2006). Similarly, studies have also implicated the hippocampus in the extinction of drug-related behaviors. Electrical stimulation of the ventral subiculum of the hippocampus reinstates cocaine-seeking (Vorel et al., 2001), and inactivation of this region abolishes cocaine drug-seeking (Sun et al., 2005). Neuronal activity within the CA1 and dentate gyrus (DG) has also been shown to change with extinction training of cocaine-associated cues providing further evidence that plasticity within this structure is associated with extinction behavior (Neisewander et al., 2000).

### Hypothalamus

A less investigated structure that has recently been implicated in extinction behavior is the hypothalamus. This structure has traditionally been shown to be involved in reward and feeding but its influence on drug-seeking behavior is becoming better understood (for reviews, see Millan et al., 2011; Marchant et al., 2012). The medial dorsal hypothalamus (MDH) is associated with the termination of motivated behaviors and, therefore, is a logical candidate for involvement in extinction learning. In rats trained to self-administer alcohol and then exposed to extinction training, infusion of the inhibitory neuropeptide known as cocaine and amphetamine-regulated transcript (CART) into the MDH prevented the expression of extinction (Marchant et al., 2010). It is important to note that a similar effect was found with the extinction of sucrose-seeking behavior suggesting the mechanisms within the LDH that help regulate extinction may not be unique to drug reinforcers (Millan et al., 2011). To add further support for the role of the MDH in extinction behavior, this region receives extensive projections from the IfL cortex (Thompson and Swanson, 1998; Heidbreder and Groenewegen, 2003). In rats exposed to extinction training after a history of alcohol administration, the expression of extinction is associated with induction of c-Fos expression in retrograde labeled IfL cortical neurons projecting to the MDH (Marchant et al., 2010; Millan et al., 2011). Together, these findings suggest plasticity-related changes in the MDH, and through its connections with the IfL cortex, can mediate the extinction of reward-seeking behavior. These results also identify a brain region to investigate as a novel candidate for the facilitation of extinction behavior.

Based on findings detailed in the preceding sections, there are several key brain regions involved in extinction behavior. The exact details of how these structures interact to form a neurocircuitry that mediates extinction behavior have yet to be fully established. However, converging lines of evidence indicate that subregions of the PFC (and their corresponding projections to subcortical structures) play a major role in the extinction of drug and fear behaviors. Peters et al. (2009) proposed that extinction of drug memories comprises overlapping neural circuitry with that of fear memories. According to the model of the neurocircuitry of fear conditioning, the PrL cortex sends excitatory projections to the BLA that, in turn, promote the expression of conditioned fear via excitation of the CeA. In contrast, the IfL cortex sends excitatory projections to GABAergic inhibitory neurons in the intercalated (ITC) cell masses in the amygdala. This leads to inhibition of the CeA and attenuation of the expression of conditioned fear, and promotes the expression of extinction behavior. In the neurocircuitry of the extinction of drug memories, the PrL cortex also sends excitatory projections to the core region of the NAc where it has been shown to regulate the expression of cocaine-seeking behavior. In contrast, excitatory projections from the IfL cortex to the shell region of the NAc promote the extinction of cocaine-seeking behavior. This proposed circuitry for the extinction of drug behaviors is depicted in Figure 3. What is currently unknown is how structures such as the BLA, hippocampus, and MDH contribute to the established role of the PFC subregions in extinction behavior.

## Glutamatergic Mechanisms in Extinction

In recent years, a number of studies have provided a more detailed analysis of the plasticity-related mechanisms that may mediate extinction behavior. Pathways connecting the various brain regions involved in extinction may differentially modulate the expression of drug-seeking and extinction of drug-seeking behavior. For instance, it was observed that there is increased activity of ventromedial PFC neurons in response to presentation of cocaine-related cues during extinction training. Interestingly, when activity in this region was inhibited, there was a corresponding decrease in extinction responding (Koya et al., 2009). Additionally, it has been shown that prefrontal regions have the ability to influence activity in other extinction-related brain structures. For instance, stimulation of IfL cortical output results in an inhibition of pyramidal neurons in the PrL cortex through a feed-forward mechanism (Ferrante et al., 2009). Similar results were found in a study that utilized optogenetic procedures to activate or inhibit specific cell types in isolated brain regions in combination with single-unit recordings of neuronal activity. It was revealed that optogenetic stimulation of viral vector encoding channel rhodopsin 2 (ChR2) excitatory neurons in the IfL cortex produced excitation of IfL cortical pyramidal neurons and also increased their responsiveness to excitatory input from multisensory brain regions (Ji and Neugebauer, 2012). It was further observed that activation of the IfL cortex inhibits PrL output, supporting the suggestion that IfL cortex mediated extinction mechanisms may involve inhibition of PrL cortex output that would ultimately mediate fear expression and possibly drug-seeking. Previous research has also shown that stimulation of the PrL region results in excitation of BLA neurons (Likhtik et al., 2005) and stimulation of the IfL region reduced the responsiveness of CeA neurons to inputs from the insula and BLA (Quirk et al., 2003). While these studies did not directly address extinction of fear expression or drug-seeking behavior, they provide support for how the IfL region of the PFC-through its direct projections to subcortical regions (e.g., amygdala, NAc, and hippocampus)-can mediate extinction behavior. Additionally, the ability of IfL cortical activation to exert inhibitory control over output from pyramidal neurons in the PrL cortex may also impact the expression of fear and drug-seeking behaviors.

The highly persistent nature of drug- and fear-related cues to induce relapse and the ineffectiveness of behavioral therapies to reduce the impact of these cues has led to a focus on understanding the neural mechanisms involved in relapse with the goal that they may be targeted as a means to enhance extinction learning. Studies have pharmacologically manipulated cellular process and substrates in specific brain regions in an attempt to “strengthen” inhibitory learning formed during extinction training. Using various behavioral paradigms such as fear-conditioning procedures and drug-self administration, investigators have begun to uncover plasticity-related mechanisms that facilitate extinction learning. Given the importance of glutamatergic transmission in learning and memory processes, a strong focus has been placed on targeting glutamate-related processes in extinction learning. Manipulation of both ionotropic and metabotropic receptors facilitates the extinction of fear-conditioning and drug-seeking behavior (for reviews, see Cleva et al., 2010; Myers et al., 2011). While blockade of NMDA receptors impairs extinction learning, enhancement of these receptors with the NMDA partial agonist d-cycloserine (DCS) facilitates the acquisition of extinction of conditioned fear and drug-seeking behavior (Myers and Carlezon, 2012). Similarly, modulation of AMPA receptor activity, which like NMDA receptors is also critically involved in synaptic plasticity, can also facilitate extinction learning (Kaplan and Moore, 2011; Myers et al., 2011).

In addition to targeting ionotropic glutamate receptors, activation of mGluR5 have been shown to facilitate extinction learning through a process that may involve enhanced NMDA receptor function. Systemic administration of the mGluR5 positive allosteric modulator CDPPB facilitates extinction of cocaine-seeking behavior in CPP (Gass and Olive, 2009) and self-administration (Cleva et al., 2011) paradigms, but does not alter the extinction of methamphetamine self-administration (Widholm et al., 2011). Further implicating mGluR5 in extinction, studies in mGluR5 knockout mice revealed marked deficits in both contextual and auditory fear extinction (Xu et al., 2009). Additionally, inhibition of mGluR5 prior to extinction learning prevented the recall of extinction learning while localized infusion of a mGluR5 antagonist in the IfL cortex produced a similar effect (Fontanez-Nuin et al., 2011). A recent study also highlighted the importance of group 1 mGluRs in the ventromedial PFC in the extinction of cocaine-seeking behavior. In rats trained to self-administer cocaine, infusion of a mGluR1/5 antagonist into the dorsomedial PFC failed to alter the rate of extinction. In contrast, infusion of a mGluR1/5 agonist had a facilitating effect on extinction of cocaine-seeking behavior (Ben-Shahar et al., 2013). This study also revealed that animals displaying deficits in extinction learning also had a significant reduction in group 1 mGluR function in the ventromedial PFC. Together these intriguing findings provide further support for glutamate-related plasticity in the IfL cortex in extinction learning.

Studies of conditioned fear have shown that inactivation of the rostral BLA (rBLA) slows cocaine cue extinction learning, and it has been suggested that simultaneous activity in the rBLA and hippocampus might be required for the acquisition of cocaine cue extinction learning (Szalay et al., 2011). Another study has shown that inactivation of the BLA not only resulted in a delay in extinction recall of an opiate reward memory, but also caused an increase in the spontaneous firing of neurons in the PrL cortex (Sun and Laviolette, 2012). This suggests that a functional link between the PrL cortex and BLA might modulate the processing of an opiate-related memory. An influence of AMPA receptor activity in the BLA during the extinction of cocaine-seeking behavior has also been reported. It was observed that expression of AMPA receptor subunit GluA1 decreased in the BLA but increased in the ventromedial PFC in response to extinction training (Nic Dhonnchadha et al., 2013), adding further support for a functional connection between the mPFC and amygdala in the extinction of drug-seeking behavior. In the hippocampus, extinction of a morphine-conditioned context was associated with changes in the phosphorylation of AMPA receptors at hippocampal synapses while no changes were observed in animals that were not exposed to extinction training (Billa et al., 2009). Furthermore, suppression of neurogenesis in the adult hippocampus after the acquisition of cocaine self-administration significantly enhanced resistance to extinction (Noonan et al., 2010). Similar to the effects observed in the rBLA, inactivation of the dorsal hippocampus slowed the rate of extinction of a cocaine memory (Szalay et al., 2011). Furthermore, cocaine self-administration training reduces neurogenesis in the DG, an effect that was normalized by extinction training (Deschaux et al., 2012). It was also observed that low frequency stimulation of the hippocampus prevented this extinction-induced normalization of DG neurogenesis. Together, these studies indicate a critical role of plasticity-related changes within the amygdala and hippocampus in the extinction of drug-seeking behavior. Although it has yet to be explored, it is possible that pharmacological manipulation of plasticity within these brain regions could serve to facilitate extinction of conditioned drug-seeking behavior.

## Noradrenergic Mechanisms in Extinction

While glutamate-related neurochemical processes have received the most attention in extinction behavior, an emerging area of interest is the role that noradrenergic mechanisms play in extinction learning (for an extensive review, see Mueller and Cahill, 2010). Norepinephrine has been shown to be involved in various aspects of memory, most notably the strengthening of memory formation (McGaugh, 2004). While there has been a renewed interest in the ability of noradrenergic mechanisms to mediate fear extinction, the results have been inconsistent. For example, it has been shown that systemic administration of the beta-adrenergic antagonist propranolol prior to extinction training impaired subsequent retrieval of contextual fear extinction (Ouyang and Thomas, 2005). However, direct infusions of norepinephrine into the amygdala after extinction training facilitated the extinction of contextual fear (Berlau and McGaugh, 2006), suggesting that noradrenergic mechanisms may help mediate the consolidation of extinction learning. It has also been shown that arousal-related norepinephrine release in the IfL cortex is important for the formation of fear extinction memory (Mueller et al., 2008).

There have been several interesting observations regarding the influence of noradrenergic mechanisms on the extinction of drug-seeking behavior. Yohimbine, an alpha2-receptor antagonist that promotes the release of norepinephrine, impairs the extinction of cocaine CPP (Davis et al., 2008) and slows the rate of extinction of cocaine self-administration (Kupferschmidt et al., 2009). Furthermore, infusion of the beta-receptor agonist clenbuterol into the IfL cortex facilitates extinction of cocaine-seeking behavior (LaLumiere et al., 2010). These studies add support to the growing body of evidence that areas of the PFC are heavily involved in extinction behavior, and one possible mechanism could be noradrenergic-related changes in this region. Norepinephrine release alters the cellular properties of target neurons that may enhance excitability and synaptic plasticity and thus promote the formation of an extinction memory (Mueller and Cahill, 2010). Support for this comes from studies showing that norepinephrine enhances intrinsic excitability in the IfL cortex (Barth et al., 2007; Mueller et al., 2008), amygdala (Tully et al., 2007), and hippocampus (Pedreira and Maldonado, 2003).

## Epigenetics and Extinction

Epigenetic mechanisms associated with extinction learning have received substantial attention over the pass several years and are providing unique insight into plasticity-related mechanisms of extinction. Epigenetic modification refers to the structural adaptation of chromosomes that results in altered activity states (Bird, 2007; Graff and Tsai, 2013). Epigenetic mechanisms exert lasting control over gene expression without altering the genetic code and may mediate stable changes in brain function (Tsankova et al., 2007). Investigation into the epigenetic regulation of neurobiological adaptations that are associated with psychiatric disorders, including addiction and PTSD, could provide novel approaches to the mechanisms underlying extinction learning.

The formation of long-term memories is thought to correlate with changes in gene expression. Research suggests that epigenetic-related mechanisms, such as histone acetylation/deacytylation and DNA methylation/demethylation, may mediate some of these processes (for a review, see Tsankova et al., 2007). For example, memory deficits in rodents can be recovered with administration of a histone deacetylase (HDAC) inhibitor, while conditioning in rodents is associated with histone protein H3 phosphoacetylation and chromatin remodeling (Levenson and Sweatt, 2005). Furthermore, synaptic plasticity is associated with epigenetic changes and can be promoted with HDAC inhibitors (Levenson et al., 2004). While these data indicate that epigenetic mechanisms are involved during the acquisition of conditioning, evidence also indicates that these same mechanisms may play a role in extinction learning.

In fear conditioning, it has been shown that acetylation and deacetylation of histones can enhance memories formed during conditioning and extinction behavior (Levenson et al., 2004; Bredy et al., 2007; Lattal et al., 2007). The non-selective HDAC inhibitor valproic acid can facilitate not only the acquisition and extinction of conditioned fear, but also the reconsolidation of this memory (Bredy and Barad, 2008). Similar results have been obtained with the HDAC inhibitor vorinostat (Fujita et al., 2012). It has also been shown that deficits in the extinction learning of conditioned fear in isogenic 129S1 (S1) mice can be recovered by administration of an HDAC inhibitor (Whittle et al., 2013). Administration of another non-selective HDAC inhibitor sodium butyrate (NaB) has a facilitating effect on the extinction of a fear memory in mice (Itzhak et al., 2012), which might be due, at least in part, to epigenetic-related mechanisms in the hippocampus and IfL cortex (Stafford et al., 2012). Furthermore, overexpression of HDAC1 in the hippocampus has also been shown to facilitate the extinction of contextual fear memories, and this effect can be prevented by inhibition of HDAC1 (Bahari-Javan et al., 2012). Finally, inhibition of p300 (a histone acetyltransferase) in the IfL cortex can enhance extinction of fear conditioning in mice, which was suggested to result from the influence of p300 on LTP in this brain region (Marek et al., 2011).

While there have been substantially fewer studies examining the epigenetic changes that accompany the extinction of drug-seeking behavior, similar results to the fear-conditioning literature have been observed. Malvaez et al. (2010) examined the effect of HDAC inhibition on the extinction of a cocaine-induced CPP. They found that systemic administration of NaB facilitated the extinction of the cocaine memory and attenuated reinstatement of cocaine-seeking behavior. Importantly, these behavioral effects correlated with enhanced acetylation of histone H3 in the NAc. Systemic administration of the HDAC3 inhibitor RGFP966 also facilitates the extinction of a cocaine-related memory, and it was suggested that this effect was mediated by enhancement of memory consolidation during extinction learning (Malvaez et al., 2013). These effects were also associated with histone acetylation linked to gene expression in the IfL cortex, hippocampus, and NAc. Taken together, observations from the fear and addiction fields have provided intriguing insights into the possible therapeutic targets related to epigenetics that could potentially be utilized to facilitate the extinction of emotionally salient memories. While further research is needed to fully clarify the roles of these mechanisms in the extinction of drug-related memories, this is a promising area of investigation for the extinction of drug cues given the established role of epigenetic mechanisms in memory.

## Extinction Versus Reconsolidation

The widely held belief that extinction learning involves the acquisition of new memories has been challenged recently with the idea that behavior typically interpreted as extinction learning may actually represent *reconsolidation* of previously formed memories (for reviews on this topic, see Dudai and Eisenberg, 2004; Nader and Einarsson, 2010; Sorg, 2012). During the initial coding of events, memories are labile, but subsequently consolidate into long-term storage through protein synthesis-dependent mechanisms (Quirk et al., 2010). Thus, extinction training may serve to reverse or update previously formed contingencies (Sorg, 2012). As such, exposure to extinction training shortly after reactivation of a fear memory attenuates recovery, renewal, and reinstatement of conditioned fear (Monfils et al., 2009; Quirk et al., 2010). Importantly, studies have shown that timing of the CS presentation is critical in order to temporarily activate the labile state in which updates to the CS-US association can occur. Reconsolidation typically requires short presentations of the CS (Nader and Hardt, 2009), and presentation of the CS alone within 6 h after memory reactivation results in behavioral effects that reflect unlearning as opposed to the inhibition of fear (Nader et al., 2000; Quirk et al., 2010). Theoretically, the ability to modify existing memories, as opposed to creating new inhibitory associations through the facilitation of extinction learning, could be advantageous over extinction-based exposure therapies. Studies show that while extinction learning can be facilitated pharmacologically, these effects can be context-dependent (Bouton, 2000, 2002, 2004; Milad et al., 2005; Woods and Bouton, 2006). Modification of the original memory, rather than the creation of competitive memories, might manifest a behavior that is more resistant to the influence of context (Quirk et al., 2010), an idea that has clinical support. For instance, administration of a beta-adrenergic receptor antagonist during reconsolidation removes the fear-arousing aspects of the conditioned memory (Soeter and Kindt, 2011). This effect was not specific to the initial stimuli used in the fear-conditioning paradigm and generalized to related stimuli. While there is excitement in the field that revolves around the influence of reconsolidation on extinction behavior, more research is clearly needed to fully elucidate the contributions of both processes in the inhibition of behavior.

## Conclusion

**Figure 4:**
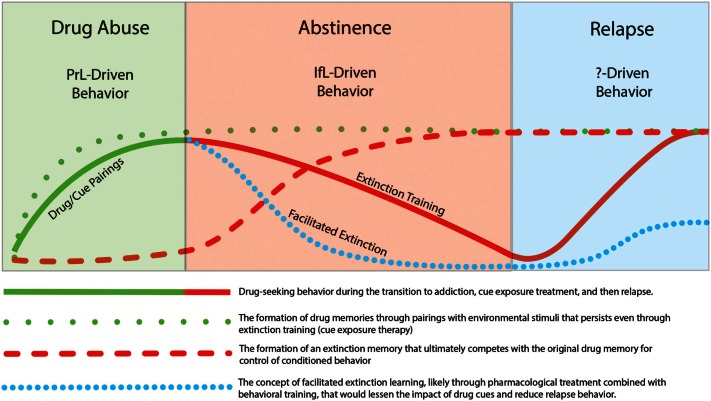
**Illustration showing that while behavioral extinction training can reduce drug-seeking behavior, the persistence of the original drug memory can promote subsequent relapse**. However, pharmacological facilitation of the extinction process may promote a stronger and more persistent extinction memory that may lead to reductions in the rate of relapse.

In this review, we focused on studies that incorporate learning principles in extinction training with the goal of lessening the influence of these cues on addictive behavior. It has been widely recognized that drug use and relapse are strongly cue specific (Drummond and Glautier, 1994) and one of the most important factors that contributes to relapse is the impact of drug cues on drug-seeking behavior. In recent years, there has been increasing attention on the neural mechanisms that underlie extinction learning in an effort to manipulate and possibly enhance learning that occurs during inhibitory conditioning. Clinically, extinction-based behavioral therapies have generally proven ineffective for suppression of relapse to drug taking. This lack of efficacy may relate to the fact that extinction learning does not erase the original drug memory but instead involves formation of a new extinction memory that acts in competition for control of behavior with the drug memory. However, the intransigent nature of the drug-memory appears to promote subsequent relapse to drug-taking. The temporal relationship of extinction and relapse are depicted in Figure 4. While extinction training alone can initially reduce drug-seeking behavior, these effects are likely context-specific. Thus, when the addict is exposed to drug cues outside of the treatment environment, the drug memory that was suppressed but not erased during extinction training, can reinitiate drug-seeking and drug use. Although speculative, pharmacological facilitation of extinction learning may enhance formation of an inhibitory memory that is much “stronger” than the initial drug memory and may help protect against cue-induced relapse. Recent research has shed light on pharmacologically targeting glutamatergic, adrenergic, and epigenetic mechanisms to enhance inhibitory learning during extinction training. Furthermore, while the neurocircuitry of extinction likely involves a distributed network of different brain regions that include the mPFC, NAc, amydala, hippocampus, and hypothalamus, recent studies have implicated opposing roles of the PrL and IfL subregions of the PFC in the control of drug-related behavior. A model has emerged in which drug-seeking is likely a PrL cortex driven behavior while extinction learning and the resulting inhibition of drug-seeking is a IfL cortex driven behavior. One aim of future research is to elucidate the contribution of these different neural regions and mechanisms to the facilitation of extinction learning to ultimately develop more effective treatments for addiction.

Although there have been substantial advances in our understanding of the neural mechanisms involved in the extinction of drug-related memories, a number of important issues need to be addressed by additional studies in the field of drug addiction. For example, while the neural circuits that mediate extinction of fear behavior do not overlap directly with those in drug-seeking behaviors, are the mechanisms that mediate extinction the same for all drugs of abuse? There is strong evidence for involvement of the PrL cortex in cocaine-seeking and IfL cortex in cocaine extinction behavior. However, there are few and sometimes conflicting findings with other drugs of abuse, such as heroin (Rogers et al., 2008), methamphetamine (Rocha and Kalivas, 2010), and alcohol (Millan et al., 2010). In addition, as recent research begins to highlight the importance of other structures in the extinction of drug memories, how do they interact with the established role of the PFC in mediating extinction behavior? The identification of the specific roles of the hippocampus, amygdala, and hypothalamus and their influence on a “final common pathway” through the PFC could provide insight into possible therapeutic targets to enhance extinction learning.

The standard procedure for extinction training is repeated presentations of the CS in absence of the US. While this method has permeated the literature since the days of Pavlov, it is not clear whether this is the most effective approach. It is of interest that several studies have examined the “retrieval-extinction” approach that combines extinction training with brief drug-memory retrieval (to activate the labile state of the memory) that have produced encouraging results (Hutton-Bedbrook and McNally, 2013).

With an increased focus on the importance of consolidation in promotion of extinction learning, a particularly interesting area of future research will be to understand the effect of sleep and sleep insomnia in extinction learning. Coordinated activity in the PFC and hippocampus during sleep is critical for the consolidation of memories (Euston et al., 2007). Sleep has been shown to promote retention of fear extinction memory (Pace-Schott et al., 2009, 2012). Interestingly, while extinction training can attenuate sleep disturbances (Wellman et al., 2008), the bidirectional relationship between these two processes and how they contribute to the extinction of drug memories is largely unexplored.

Lastly, there are multiple studies showing that context is a major hurdle in using extinction-based treatment approaches, and another important area of research will be to determine whether context specificity of extinction can be prevented. Context is not limited to common environmental stimuli associated with drug use and can include factors such as drug states and the passage of time (Bouton et al., 2012). Thus, there are many types of stimuli that serve as contextual cues to promote relapse, and future research is needed in order to understand how pharmacological manipulation of extinction training could be used to minimize the influence of context.

## Conflict of Interest Statement

The authors declare that the research was conducted in the absence of any commercial or financial relationships that could be construed as a potential conflict of interest.
